# National Health Work for Women

**Published:** 1917-07-28

**Authors:** 


### NATIONAL HEALTH WORK FOR WOMEN.

August will probably, even under present conditions, be something of a holiday month for all who can make it so, but those who plan to take up work in the autumn should be deciding now on their aim and fixing their engagements so as to waste no time on their return. During the coming months there will be many fresh developments in work for women, and of these many will be blind alleys, temporary jobs which will end with the necessities of the war, or with the return of the man whose post is being occupied. At the same time the need that a greater number of upper and middle class women shall earn their own living will become more urgent. It is, therefore, very desirable that offers of sound training for a permanent, well-paid, and nationally useful profession should be made known as widely as possible.

The profession, or rather group of professions, included under the heading of National Health Work offers now a very special opportunity, and one which should appeal to a number of women who regret that they did not earlier take up the profession of nursing. Now, it may be, they are past the ordinary age, and cannot well spare time for a prolonged training. But the woman in her "second youth" has advantages over the girl in health work. Every item of experience she has gained in social service or in teaching or in family life or in reading and thinking adds to her equipment, and the great and growing movement in which she can take part welcomes initiative, enterprise, and originality, or at least freshness of thought and method, while its approaching co-ordination into an accepted department of national life demands capacity to receive and profit by a sound and thorough training. No one knows better than the voluntary worker that the day of amateur work is past.

It is not possible, of course, for every worker to come to London for training. Our great cities and the younger universities have formulated some schemes on good lines that are constantly being improved. But those who can settle in London in September and undergo the courses of training offered either by the National Health Society, 53 Berners Street, W., or by the Royal Sanitary Institute, 90 Buckingham Palace Road, S.W., may fit themselves, at a very moderate cost in time and money, for excellent and increasingly numerous appointments.

The National Health Society's latest offer is a special training course for ladies desiring to become sanitary inspectors, sitting for examination by the board appointed by the Local Government Board. This course lasts twelve or thirteen weeks, beginning in September and again in January. A longer course, beginning in September and lasting six months, leads up to the Society's own well-recognised diploma. The Local Government Board gives special recognition to this qualification under its Health Visitors (London) Order. The examination is held in May. Students are advised to add a three months' hospital training, if possible, or the C.M.B. diploma, as these qualifications are most valuable. Or both courses may be taken; some of the lectures, etc., are common to both, and if this is done the fee for the composite course is £21; for Course I. it is £12 12s., for Course II. £15 15s. Some of the posts taken are, for instance, those of health visitor, lady almoner, relieving officer, and other posts under Guardians and medical officers, sanitary inspector, superintendent of mother-school or other institution, health lecturer, welfare worker. The study consists of lectures, tutorial study, and visits and demonstrations at all kinds of work centres.

The certificate of the Royal Sanitary Institute is equally recognised by the Local Government Board among qualifications for health visitors, and their offer of a number of short courses for the autumn term is set forth in a very useful booklet, which may be had from the office. Those who cannot afford the time or cost for the thorough training of the National Health Society, or who wish to add to qualifications already possessed, will find excellent help here. Demonstrations are given in the Parkes Museum and elsewhere. We should like to draw special attention to the course beginning October 8, at the hour of six, convenient for busy women, at the low fee of £1 11s. 6d. for twenty-one lectures, by experts in each subject, besides demonstrations and liberty to attend six infant consultations directed by Dr. Eric Pritchard. Such varied subjects as school buildings, house sanitation, the care of infants and children, infectious, tuberculous, and venereal disease, enter into this course, which may very profitably be combined with another designed for maternity and child-welfare workers, but equally profitable to any women engaged in any sort of public service, or even wishing to understand the problems of the hour. Legislation governing the lives of women and children is usefully included in the subjects. The fee for this course is also £1 11s. 6d., or the two combined £2 12s. 6d., with 10s. 6d. carried forward towards fee for examination if this is taken.

